# μ-Oxido-bis{[2,2-bis­(3,5-dimethyl-1*H*-pyrazol-1-yl)acetato-κ^3^ *N* ^2^,*O*,*N* ^2′^]chloridooxidomolybdenum(V)} mono­hydrate

**DOI:** 10.1107/S1600536810045393

**Published:** 2010-11-24

**Authors:** Amalija Golobič, Boris Čeh

**Affiliations:** a Faculty of Chemistry and Chemical Technology, University of Ljubljana, Aškerčeva 5, 1000 Ljubljana, Slovenia

## Abstract

In the binuclear title compound, [Mo_2_(C_12_H_15_N_4_O_2_)_2_Cl_2_O_3_]·H_2_O, the complex mol­ecules have approximate *C*_2_ symmetry. Both Mo^V^ atoms have a distorted octa­hedral coordination environment with *cis*-positioned terminal chloride and oxide groups. The heteroscorpionate organic ligand binds to the Mo^V^ atom *via* an N_2_O donor set. The water mol­ecule bridges two complex mol­ecules, forming O—H⋯O and O—H⋯Cl hydrogen bonds to the acetate group and to the chloride ligands.

## Related literature

The prepraration of the first ‘scorpionate’ complex was described by Trofimenko (1967 ▶). For the importance of the structures of Mo(VI/V/IV) complexes related to the Mo-enzymes, see: Hille (1996 ▶); Heinze & Fischer (2010 ▶). For complexes with κ^3^ *N*,*N*′,*O*-bound heteroscorpionate ligands, see: Otero *et al.* (2004 ▶); Burzlaff (2008 ▶); Kitanovski *et al.* (2006 ▶). For Mo complexes with bis­(3,5 dimethyl-1*H*-pyrazol-1-yl)acetate ligands, see: Kitanovski *et al.* (2006 ▶); Hammes *et al.* (2004 ▶). For the weighting scheme used in the refinement, see: Wang *et al.* (1985 ▶)
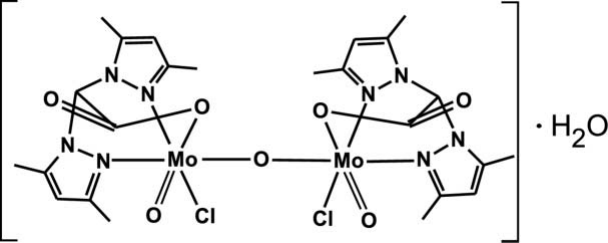


## Experimental

### 

#### Crystal data

[Mo_2_(C_12_H_15_N_4_O_2_)_2_Cl_2_O_3_]·H_2_O
*M* *_r_* = 823.36
Orthorhombic, 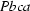

*a* = 14.6869 (1) Å
*b* = 20.6499 (2) Å
*c* = 21.0082 (2) Å
*V* = 6371.43 (10) Å^3^
*Z* = 8
Mo *K*α radiation
μ = 1.01 mm^−1^
*T* = 293 K
0.30 × 0.15 × 0.05 mm

#### Data collection

Nonius KappaCCD diffractometer
Absorption correction: multi-scan *DENZO-SMN* (Otwinowski & Minor, 1997) ▶ *T*_min_ = 0.69, *T*_max_ = 0.95
91066 measured reflections
7300 independent reflections
5534 reflections with *I* > 2σ(*I*)
*R*_int_ = 0.064

#### Refinement

*R*[*F* ^2^ > 2σ(*F* ^2^)] = 0.038
*wR*(*F* ^2^) = 0.028
*S* = 1.42
6640 reflections
397 parameters
H-atom parameters not refined
Δρ_max_ = 0.97 e Å^−3^
Δρ_min_ = −1.50 e Å^−3^

### 

Data collection: *COLLECT* (Nonius, 2000 ▶); cell refinement: *DENZO-SMN* (Otwinowski & Minor, 1997 ▶); data reduction: *DENZO-SMN*; program(s) used to solve structure: *SIR97* (Altomare *et al.*, 1999 ▶); program(s) used to refine structure: *Xtal3*.6 (Hall *et al.*, 1999 ▶); molecular graphics: *ORTEP-3* (Farrugia, 1997 ▶); software used to prepare material for publication: *Xtal3*.6.

## Supplementary Material

Crystal structure: contains datablocks global, I. DOI: 10.1107/S1600536810045393/gk2307sup1.cif

Structure factors: contains datablocks I. DOI: 10.1107/S1600536810045393/gk2307Isup2.hkl

Additional supplementary materials:  crystallographic information; 3D view; checkCIF report

## Figures and Tables

**Table 1:** Selected bond lengths (Å)

Mo1—Cl1	2.3594 (11)
Mo1—O1	1.675 (3)
Mo1—O1a	2.151 (3)
Mo1—O3	1.865 (3)
Mo1—N2a	2.225 (3)
Mo1—N2b	2.201 (3)
Mo2—Cl2	2.3759 (11)
Mo2—O1c	2.150 (3)
Mo2—O2	1.674 (3)
Mo2—O3	1.861 (3)
Mo2—N2c	2.232 (3)
Mo2—N2d	2.190 (3)

**Table 2:** Hydrogen-bond geometry (Å, °)

*D*—H⋯*A*	*D*—H	H⋯*A*	*D*⋯*A*	*D*—H⋯*A*
O1w—H1w⋯O2c	1.02	2.23	2.889 (8)	121
O1w—H2w⋯Cl2^i^	1.00	2.42	3.335 (7)	151

Symmetry code: (i) 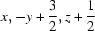.

**Fig. 1.**
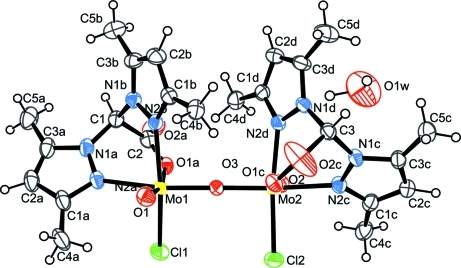
ORTEP drawing of the asymmetric unit of the title compound, showing the atom-labelling scheme. Displacement ellipsoids are drawn at the 40% probability level and H atoms are drawn as small spheres of arbitrary radii.

## supplementary crystallographic information

### Comment

Since the preparation of the first tris(pyrazolyl)borate-complexes by Trofimenko (1967) the coordination compounds of almost all transition elements with different "scorpionate" ligands have been prepared. The characteristics and the synthetic routes of divers tripodal heteroscorpionate N,*N*,*O*-, N,*N*,*S*- and, *N*,*N*,*N*- ligands based on bis(pyrazol-1-yl)acetate, -thioacetate and -ethoxide with pyrazolyl rings substituted at 3 and 5 positions, as well as their complexes with different metals have been discussed. (Otero *et al.*, 2004). For some time afterwards, the complexes with metal atoms coordinated with tripodal κ^3^*N*,*N*',*O*-bound "scorpionate" ligands have attracted considerable interest because they can serve as structural models, mimicking the active sites like, for example, the 2-His-1-carboxylate triad, which is present in different metalloenzymes and –proteins, mostly containing Zn, Fe, Mn, Ni, Co and Mo atoms (Burzlaff, 2008). The mononuclear molybdenum-containing enzymes serve for catalyzing of a net oxygen atom transfer with the Mo atom cycling between +4 and +6 oxidation states (Hille, 1996). The elucidation of the structures of mononuclear Mo(VI/V/IV) complexes can help the understanding of interaction of the intermediate, and resting states of these enzymes (Heinze & Fischer, 2010). The complexes with di-1*H*-pyrazol-1-ylacetate, substituted at the 3 and 5 positions, are known with more than a half of d-elements in different oxidation states (Kitanovski *et al.*, 2006). Some Mo(VI), and Mo(V) complexes with bdmpza as ligand have already been prepared so far (Hammes *et al.*, 2004; Kitanovski *et al.*, 2006).

The compound crystallizes in the orthorhombic space group Pbca with eight binuclear complex molecules and eight water molecules per unit cell. Both MoCl(O)(bdmpza) moieties are symmetry independent. The Mo1—-O1 and Mo2—-O2 bond lengths are 1.675 (3) and 1.674 (3) Å, and the Mo1—-Cl1 and Mo2—-Cl2 bond distances are 2.3594 (11) and 2.3759 (11) Å, respectively. With respect to the nonlinear Mo—-O—-Mo bridge (178.31 (16)°), the Mo=O vectors in the binuclear unit adopt an anti-orientation (torsion angle O1—Mo1—Mo2—O2 is 175.59 (14)°), and the Mo—-Cl vectors an approximate *cis*-orientation (torsion angle Cl1—Mo1—Mo2—Cl2 is -31.01 (4)°). The O-atom of Mo=O and the coordinated O-atom of the acetate group are in *trans*-position (O1—Mo1—O1a 164.75 (12) and O2—Mo2—O1c 165.25 (12)°). Both central atoms have a significantly distorted octahedral coordination, caused in first line by a typically low angles between κ^3^*N*,*N*',*O*-coordination bonds with Mo-atom (between 78.28 (11) and 81.07 (12)° for Mo1, and between 78.09 (10) and 80.90 (11)° for Mo2). The high values are also observed between Mo=O and Mo—-Cl bonds (102.86 (10)° for Mo1 and 102.51 (10)° for Mo2, respectively). The solvate water acts as a donor of two weak hydrogen bonds accepted by the uncoordinated O2c of the acetate ligand from the same asymmetric unit (with O1w···O2c distance 2.889 (8) Å) and Cl2 from symmetry related unit (with O1w···Cl2(*x*,3/2 - *y*,1/2 + *z*) distance 3.335 (7) Å).

### Experimental

A mixture of MoCl_4_(CH_3_CN)_2_ (0.450 mg, 1.40 mmol), Hdmpza (0.347 g, 1.40 mmol) and acetonitrile (20 ml) was stirred at room temperature. At first the mixture became clear and after an hour the orange precipitate started to separate. After the filtration of the precipitate, a small amount of water (0.13 g) was added to the portion of filtrate (0.65 g) and the solution left on air at room temperature. After about 14 h the black crystals, suitable for X-ray diffraction started to grow and were isolated in 45% yield. Anal. Calcd. for C_24_H_32_Cl_2_N_8_O_8_: C,35.01; H, 3.92; N, 13.61. Found: C, 35.74; H, 4.03; N, 13.71.

### Refinement

Full matrix least-squares refinement on F values with anisotropic displacement parameters for all non-hydrogen atoms was employed. Hydrogen atoms were located from difference Fourier maps. Their parameters were not refined. A REGINA (Wang *et al.*, 1985) weighting scheme using the normal equation of the second order was applied for individual reflections so that w= A(0,0) + A(1,0)V(F) + A(0,1)V(S) + A(2,0)V(F)^2^ + A(0,2)V(S)^2^ + A(1,1)V(F)V(S), where V(F)= F_obs_/F_obs_(max), F_obs_(max)= 496.47 and V(S)=(sinθ/λ)/ ((sinθ/λ)(max)),(sinθ/λ)(max)=.6495. The parameters were: A(0,0) = 110.7607, A(1,0) = .7072179 A(0,1) = -502.5041, A(2,0) = -.0004053 A(1,1) = -1.637116, A(0,2) = 576.1985. The location of the deepest hole is at the site of Mo2 atom.

### Crystal data

**Table:**

[Mo_2_(C_12_H_15_N_4_O_2_)_2_Cl_2_O_3_]·H_2_O	*F*(000) = 3312
*M**_r_* = 823.36	*D*_x_ = 1.717 Mg m^−^^3^
Orthorhombic, *P**b**c**a*	Mo *K*α radiation, λ = 0.71073 Å
Hall symbol: -p 2ac 2ab	Cell parameters from 7980 reflections
*a* = 14.6869 (1) Å	θ = 2.6–27.5°
*b* = 20.6499 (2) Å	µ = 1.01 mm^−^^1^
*c* = 21.0082 (2) Å	*T* = 293 K
*V* = 6371.43 (10) Å^3^	Plate, black
*Z* = 8	0.3 × 0.15 × 0.05 mm

### Data collection

**Table:**

Nonius KappaCCD diffractometer	5534 reflections with *F*^2^ > 2σ(*F*^2^)
φ and ω scans	*R*_int_ = 0.064
Absorption correction: multi-scan *DENZO-SMN* (Otwinowski & Minor, 1997)	θ_max_ = 27.5°, θ_min_ = 2.6°
*T*_min_ = 0.69, *T*_max_ = 0.95	*h* = −19→19
91066 measured reflections	*k* = −26→26
7300 independent reflections	*l* = −27→27

### Refinement

**Table:**

Refinement on *F*	0 constraints
Least-squares matrix: full	Primary atom site location: structure-invariant direct methods
*R*[*F*^2^ > 2σ(*F*^2^)] = 0.038	Hydrogen site location: difference Fourier map
*wR*(*F*^2^) = 0.028	H-atom parameters not refined
*S* = 1.42	A REG*I*NA (Wang et al., 1985) *w*eighting scheme using the normal equation of the second order *w*as applied for individual reflections so that *w* = A(0,0) + A(1,0)V(*F*) + A(0,1)V(S) + A(2,0)V(*F*)^2^ + A(0,2)V(S)^2^ + A(1,1)V(*F*)V(S), where V(*F*) = *F*_obs_/*F*_obs_(max), *F*_obs_(max) = 496.47 and V(S) = (sinθ/λ)/ ((sinθ/λ)(max)), (sinθ/λ)(max) = .6495. The parameters *w*ere: A(0,0) = 110.7607, A(1,0) = .7072179 A(0,1) = -502.5041, A(2,0) = -.0004053 A(1,1) = -1.637116, A(0,2) = 576.1985
6640 reflections	(Δ/σ)_max_ = 0.002
397 parameters	Δρ_max_ = 0.97 e Å^−^^3^
0 restraints	Δρ_min_ = −1.50 e Å^−^^3^

### Special details

**Table:**

Refinement. Independent reflections: contributing reflections are all observed (I > 2?(I)) and those "less than" reflections for which Fcal > Fobs

### Fractional atomic coordinates and isotropic or equivalent isotropic displacement parameters (Å^2^)

**Table:**

	*x*	*y*	*z*	*U*_iso_*/*U*_eq_	
Mo1	0.356763 (18)	0.580454 (15)	0.363344 (13)	0.03224 (16)	
Mo2	0.127609 (18)	0.634123 (13)	0.418255 (13)	0.03082 (15)	
Cl1	0.33672 (7)	0.63258 (6)	0.26439 (5)	0.0575 (6)	
Cl2	0.13854 (8)	0.72985 (5)	0.35598 (5)	0.0518 (5)	
O1	0.42851 (19)	0.63004 (14)	0.40162 (14)	0.0469 (14)	
O2	0.05836 (19)	0.58758 (15)	0.37453 (13)	0.0459 (14)	
O3	0.24283 (17)	0.60782 (13)	0.39192 (12)	0.0376 (12)	
O1a	0.29066 (17)	0.49772 (14)	0.32167 (12)	0.0403 (14)	
O1c	0.19247 (17)	0.68744 (13)	0.49357 (13)	0.0389 (13)	
O1w	0.1181 (6)	0.7030 (5)	0.7123 (3)	0.120 (5)	
O2a	0.2706 (3)	0.3928 (2)	0.3027 (2)	0.072 (2)	
O2c	0.2051 (4)	0.7262 (3)	0.5911 (2)	0.102 (3)	
N1a	0.4782 (2)	0.45992 (18)	0.32861 (16)	0.0426 (17)	
N1b	0.3950 (2)	0.44470 (16)	0.42532 (16)	0.0381 (15)	
N1c	0.0069 (2)	0.67646 (15)	0.53542 (15)	0.0364 (15)	
N1d	0.1010 (2)	0.58456 (17)	0.55781 (15)	0.0363 (15)	
N2a	0.4745 (2)	0.52597 (18)	0.32370 (15)	0.0404 (17)	
N2b	0.3723 (2)	0.50768 (15)	0.43911 (14)	0.0348 (14)	
N2c	0.0083 (2)	0.67518 (16)	0.47019 (15)	0.0374 (15)	
N2d	0.1203 (2)	0.56463 (15)	0.49686 (15)	0.0343 (14)	
C1	0.4037 (2)	0.4250 (2)	0.35926 (19)	0.0400 (17)	
C2	0.3131 (3)	0.4384 (2)	0.32399 (19)	0.042 (2)	
C3	0.0850 (2)	0.6528 (2)	0.57055 (17)	0.0363 (17)	
C4	0.1695 (3)	0.6934 (2)	0.5519 (2)	0.043 (2)	
C1a	0.5527 (3)	0.5441 (3)	0.2959 (2)	0.050 (2)	
C1b	0.3692 (2)	0.5115 (2)	0.50244 (17)	0.0373 (18)	
C1c	−0.0725 (3)	0.69854 (18)	0.4513 (2)	0.042 (2)	
C1d	0.1307 (2)	0.50069 (18)	0.5001 (2)	0.0386 (18)	
C2a	0.6050 (3)	0.4893 (3)	0.2824 (2)	0.054 (2)	
C2b	0.3862 (3)	0.4508 (2)	0.5289 (2)	0.047 (2)	
C2c	−0.1235 (3)	0.7160 (2)	0.5045 (2)	0.046 (2)	
C2d	0.1215 (3)	0.4801 (2)	0.5624 (2)	0.049 (2)	
C3a	0.5574 (3)	0.4366 (3)	0.30345 (19)	0.051 (2)	
C3b	0.4029 (3)	0.4091 (2)	0.4791 (2)	0.043 (2)	
C3c	−0.0725 (3)	0.70158 (18)	0.5574 (2)	0.041 (2)	
C3d	0.1028 (3)	0.5335 (2)	0.5988 (2)	0.046 (2)	
C4a	0.5761 (3)	0.6135 (3)	0.2839 (3)	0.064 (3)	
C4b	0.3479 (3)	0.5733 (2)	0.53618 (17)	0.0436 (19)	
C4c	−0.0982 (3)	0.7015 (2)	0.3832 (2)	0.054 (2)	
C4d	0.1487 (3)	0.46071 (19)	0.4415 (2)	0.048 (2)	
C5a	0.5788 (4)	0.3660 (3)	0.3004 (3)	0.066 (3)	
C5b	0.4230 (4)	0.3388 (2)	0.4802 (3)	0.059 (3)	
C5c	−0.0935 (3)	0.7086 (2)	0.6267 (2)	0.056 (2)	
C5d	0.0856 (4)	0.5399 (3)	0.6682 (2)	0.066 (3)	
H1	0.41320	0.37560	0.36130	0.05100*	
H3	0.07365	0.65819	0.61512	0.04400*	
H2a	0.66284	0.48881	0.26212	0.06900*	
H2b	0.37560	0.44131	0.57470	0.06000*	
H2c	−0.18830	0.72980	0.50800	0.05800*	
H2d	0.12743	0.43666	0.57739	0.04800*	
H41a	0.52780	0.63990	0.29460	0.09700*	
H41b	0.40378	0.59663	0.54040	0.03500*	
H41c	−0.16109	0.69029	0.37845	0.08300*	
H41d	0.09551	0.46090	0.41535	0.07400*	
H42a	0.62821	0.62497	0.30871	0.09700*	
H42b	0.30666	0.60026	0.51266	0.03500*	
H42c	−0.08793	0.74444	0.36759	0.08300*	
H42d	0.19870	0.47863	0.41782	0.07400*	
H43a	0.58964	0.61895	0.23938	0.09700*	
H43b	0.32447	0.56634	0.57824	0.03500*	
H43c	−0.06118	0.67138	0.35975	0.08300*	
H43d	0.16272	0.41687	0.45318	0.07400*	
H51a	0.61091	0.35634	0.26196	0.10200*	
H51b	0.47000	0.33311	0.45370	0.09200*	
H51c	−0.15547	0.72295	0.63045	0.08700*	
H51d	0.02901	0.56177	0.67437	0.08600*	
H52a	0.61542	0.35504	0.33685	0.10200*	
H52b	0.43847	0.32526	0.52219	0.09200*	
H52c	−0.08809	0.66839	0.64895	0.08700*	
H52d	0.08187	0.49797	0.68766	0.08600*	
H53a	0.52271	0.34224	0.30207	0.10200*	
H53b	0.37110	0.31483	0.46523	0.09200*	
H53c	−0.05527	0.74058	0.64650	0.08700*	
H53d	0.13301	0.56460	0.68810	0.08600*	
H1w	0.17930	0.69550	0.69110	0.13000*	
H2w	0.14750	0.72429	0.75000	0.13000*	

### Atomic displacement parameters (Å^2^)

**Table:**

	*U*^11^	*U*^22^	*U*^33^	*U*^12^	*U*^13^	*U*^23^
Mo1	0.02782 (15)	0.04180 (17)	0.02709 (16)	0.00192 (11)	0.00332 (10)	0.00246 (11)
Mo2	0.02996 (15)	0.03545 (16)	0.02706 (15)	0.00194 (11)	0.00348 (11)	0.00135 (11)
Cl1	0.0550 (5)	0.0798 (7)	0.0378 (5)	0.0089 (5)	0.0059 (4)	0.0207 (5)
Cl2	0.0681 (6)	0.0448 (5)	0.0424 (5)	0.0066 (4)	0.0105 (4)	0.0108 (4)
O1	0.0440 (13)	0.0474 (14)	0.0492 (16)	−0.0056 (12)	−0.0014 (11)	−0.0005 (12)
O2	0.0472 (13)	0.0515 (15)	0.0389 (14)	−0.0047 (12)	0.0008 (11)	−0.0033 (11)
O3	0.0364 (12)	0.0438 (12)	0.0327 (12)	0.0079 (10)	0.0077 (10)	0.0078 (10)
O1a	0.0328 (11)	0.0529 (17)	0.0351 (13)	0.0034 (10)	−0.0054 (9)	−0.0023 (11)
O1c	0.0348 (12)	0.0435 (13)	0.0384 (15)	−0.0077 (10)	0.0049 (10)	−0.0005 (10)
O1w	0.139 (5)	0.157 (6)	0.064 (3)	−0.019 (5)	−0.007 (3)	−0.032 (3)
O2a	0.071 (2)	0.0570 (19)	0.088 (3)	−0.0032 (17)	−0.036 (2)	−0.0100 (17)
O2c	0.095 (3)	0.157 (5)	0.052 (2)	−0.078 (3)	0.013 (2)	−0.035 (3)
N1a	0.0359 (15)	0.058 (2)	0.0343 (16)	0.0101 (14)	0.0017 (12)	−0.0037 (14)
N1b	0.0367 (14)	0.0422 (16)	0.0353 (16)	0.0013 (11)	0.0000 (12)	−0.0010 (13)
N1c	0.0324 (14)	0.0445 (16)	0.0324 (16)	0.0012 (12)	0.0051 (12)	−0.0025 (12)
N1d	0.0345 (13)	0.0441 (17)	0.0304 (14)	−0.0014 (12)	0.0043 (11)	0.0039 (13)
N2a	0.0309 (14)	0.058 (2)	0.0321 (16)	0.0020 (13)	0.0027 (11)	−0.0023 (13)
N2b	0.0314 (13)	0.0442 (16)	0.0288 (14)	0.0031 (12)	0.0003 (11)	−0.0010 (11)
N2c	0.0349 (15)	0.0454 (16)	0.0318 (15)	0.0022 (12)	0.0000 (12)	−0.0009 (12)
N2d	0.0288 (13)	0.0392 (16)	0.0350 (14)	−0.0010 (11)	0.0024 (11)	0.0024 (11)
C1	0.0377 (16)	0.0495 (19)	0.0328 (17)	0.0045 (15)	−0.0019 (14)	−0.0028 (16)
C2	0.0385 (18)	0.052 (2)	0.0344 (19)	−0.0014 (16)	−0.0013 (14)	−0.0053 (16)
C3	0.0345 (16)	0.0440 (19)	0.0304 (17)	0.0019 (14)	0.0025 (13)	0.0002 (13)
C4	0.0388 (18)	0.053 (2)	0.036 (2)	−0.0095 (16)	−0.0013 (16)	−0.0028 (16)
C1a	0.0338 (19)	0.083 (3)	0.034 (2)	0.002 (2)	0.0060 (15)	0.0015 (18)
C1b	0.0322 (16)	0.052 (2)	0.0278 (16)	−0.0040 (15)	−0.0030 (13)	−0.0007 (14)
C1c	0.0352 (18)	0.0388 (18)	0.051 (2)	0.0031 (14)	−0.0012 (16)	−0.0002 (15)
C1d	0.0305 (16)	0.0362 (17)	0.049 (2)	−0.0019 (14)	0.0036 (15)	0.0045 (14)
C2a	0.039 (2)	0.088 (3)	0.037 (2)	0.010 (2)	0.0073 (15)	−0.005 (2)
C2b	0.050 (2)	0.060 (2)	0.0324 (18)	−0.0022 (18)	−0.0069 (16)	0.0076 (17)
C2c	0.038 (2)	0.047 (2)	0.052 (2)	0.0073 (16)	0.0064 (17)	0.0044 (16)
C2d	0.052 (2)	0.0403 (19)	0.053 (2)	−0.0022 (17)	0.0102 (18)	0.0125 (16)
C3a	0.042 (2)	0.079 (3)	0.0312 (18)	0.017 (2)	0.0004 (15)	−0.0078 (19)
C3b	0.0430 (18)	0.047 (2)	0.039 (2)	−0.0044 (16)	−0.0104 (15)	0.0078 (16)
C3c	0.0371 (18)	0.0363 (17)	0.050 (2)	0.0014 (14)	0.0108 (16)	−0.0035 (15)
C3d	0.047 (2)	0.052 (2)	0.038 (2)	−0.0036 (16)	0.0045 (15)	0.0154 (16)
C4a	0.041 (2)	0.092 (4)	0.060 (3)	−0.011 (2)	0.0163 (19)	0.011 (2)
C4b	0.0418 (18)	0.057 (2)	0.0317 (17)	0.0051 (16)	−0.0026 (14)	−0.0077 (16)
C4c	0.046 (2)	0.065 (3)	0.051 (2)	0.0140 (18)	−0.0073 (17)	0.005 (2)
C4d	0.046 (2)	0.0378 (18)	0.060 (2)	−0.0017 (15)	0.0043 (17)	−0.0061 (16)
C5a	0.063 (3)	0.076 (3)	0.059 (3)	0.023 (2)	0.004 (2)	−0.015 (2)
C5b	0.069 (3)	0.049 (2)	0.060 (3)	0.000 (2)	−0.014 (2)	0.007 (2)
C5c	0.059 (2)	0.060 (2)	0.050 (2)	0.013 (2)	0.012 (2)	−0.0029 (19)
C5d	0.089 (4)	0.070 (3)	0.038 (2)	−0.004 (2)	0.004 (2)	0.011 (2)

### Geometric parameters (Å, °)

**Table:**

Mo1—Cl1	2.3594 (11)	C1d—C2d	1.383 (6)
Mo1—O1	1.675 (3)	C1d—C4d	1.506 (6)
Mo1—O1a	2.151 (3)	C2a—C3a	1.367 (8)
Mo1—O3	1.865 (3)	C2b—C3b	1.377 (6)
Mo1—N2a	2.225 (3)	C2c—C3c	1.373 (6)
Mo1—N2b	2.201 (3)	C2d—C3d	1.370 (6)
Mo2—Cl2	2.3759 (11)	C3—C4	1.548 (5)
Mo2—O1c	2.150 (3)	C3a—C5a	1.493 (9)
Mo2—O2	1.674 (3)	C3b—C5b	1.482 (6)
Mo2—O3	1.861 (3)	C3c—C5c	1.495 (6)
Mo2—N2c	2.232 (3)	C3d—C5d	1.486 (6)
Mo2—N2d	2.190 (3)	C1—H1	1.0305
O1a—C2	1.270 (5)	C2a—H2a	0.9504
O1c—C4	1.277 (5)	C2b—H2b	0.9942
O2a—C2	1.215 (6)	C2c—H2c	0.9962
O2c—C4	1.188 (7)	C2d—H2d	0.9547
O1w—H1w	1.0150	C3—H3	0.9575
O1w—H2w	1.0035	C4a—H42a	0.9558
N1a—N2a	1.369 (5)	C4a—H41a	0.9225
N1a—C3a	1.365 (6)	C4a—H43a	0.9628
N1a—C1	1.460 (5)	C4b—H42b	0.9597
N1b—C3b	1.353 (5)	C4b—H43b	0.9591
N1b—N2b	1.374 (5)	C4b—H41b	0.9558
N1b—C1	1.452 (5)	C4c—H43c	0.9619
N1c—C3	1.449 (4)	C4c—H41c	0.9574
N1c—N2c	1.371 (4)	C4c—H42c	0.9574
N1c—C3c	1.357 (5)	C4d—H43d	0.9603
N1d—C3	1.454 (5)	C4d—H42d	0.9611
N1d—N2d	1.375 (4)	C4d—H41d	0.9550
N1d—C3d	1.362 (5)	C5a—H52a	0.9627
N2a—C1a	1.342 (5)	C5a—H51a	0.9562
N2b—C1b	1.334 (5)	C5a—H53a	0.9595
N2c—C1c	1.341 (5)	C5b—H52b	0.9529
N2d—C1d	1.331 (5)	C5b—H51b	0.8946
C1—C2	1.548 (5)	C5b—H53b	0.9617
C1a—C2a	1.397 (8)	C5c—H52c	0.9562
C1a—C4a	1.495 (9)	C5c—H53c	0.9615
C1b—C2b	1.394 (6)	C5c—H51c	0.9604
C1b—C4b	1.493 (6)	C5d—H53d	0.9590
C1c—C4c	1.481 (6)	C5d—H51d	0.9547
C1c—C2c	1.393 (6)	C5d—H52d	0.9591
			
Cl1—Mo1—O1	102.86 (10)	N1c—C3—N1d	111.2 (3)
Cl1—Mo1—O1a	86.99 (8)	N1c—C3—C4	108.9 (3)
Cl1—Mo1—O3	91.92 (8)	N1d—C3—C4	110.4 (3)
Cl1—Mo1—N2a	89.88 (9)	N1a—C3a—C5a	122.7 (5)
Cl1—Mo1—N2b	164.08 (9)	C2a—C3a—C5a	131.0 (5)
O1—Mo1—O1a	164.75 (12)	N1a—C3a—C2a	106.3 (5)
O1—Mo1—O3	102.98 (13)	N1b—C3b—C2b	106.2 (4)
O1—Mo1—N2a	89.99 (13)	C2b—C3b—C5b	129.5 (4)
O1—Mo1—N2b	90.29 (13)	N1b—C3b—C5b	124.2 (4)
O1a—Mo1—O3	88.09 (11)	N1c—C3c—C2c	106.0 (4)
O1a—Mo1—N2a	78.28 (11)	C2c—C3c—C5c	130.9 (4)
O1a—Mo1—N2b	78.40 (11)	N1c—C3c—C5c	123.1 (4)
O3—Mo1—N2a	166.14 (12)	N1d—C3d—C2d	105.9 (4)
O3—Mo1—N2b	93.85 (11)	N1d—C3d—C5d	123.3 (4)
N2a—Mo1—N2b	81.07 (12)	C2d—C3d—C5d	130.8 (4)
Cl2—Mo2—O1c	87.09 (8)	O1c—C4—O2c	127.2 (5)
Cl2—Mo2—O2	102.51 (10)	O1c—C4—C3	113.7 (3)
Cl2—Mo2—O3	91.01 (9)	O2c—C4—C3	119.1 (4)
Cl2—Mo2—N2c	90.35 (9)	N1b—C1—H1	104.45
Cl2—Mo2—N2d	164.48 (9)	C2—C1—H1	108.26
O1c—Mo2—O2	165.25 (12)	N1a—C1—H1	113.94
O1c—Mo2—O3	88.02 (10)	C3a—C2a—H2a	126.42
O1c—Mo2—N2c	78.09 (10)	C1a—C2a—H2a	126.24
O1c—Mo2—N2d	78.60 (11)	C1b—C2b—H2b	122.36
O2—Mo2—O3	102.81 (13)	C3b—C2b—H2b	129.79
O2—Mo2—N2c	90.54 (13)	C1c—C2c—H2c	130.32
O2—Mo2—N2d	90.45 (13)	C3c—C2c—H2c	121.57
O3—Mo2—N2c	165.95 (11)	C3d—C2d—H2d	126.20
O3—Mo2—N2d	94.44 (11)	C1d—C2d—H2d	126.32
N2c—Mo2—N2d	80.90 (11)	N1c—C3—H3	108.73
Mo1—O1a—C2	129.3 (3)	N1d—C3—H3	108.72
Mo2—O1c—C4	129.7 (3)	C4—C3—H3	108.91
Mo1—O3—Mo2	178.31 (16)	C1a—C4a—H41a	110.40
H1w—O1w—H2w	91.84	C1a—C4a—H42a	109.25
N2a—N1a—C3a	110.9 (4)	H41a—C4a—H42a	109.66
C1—N1a—C3a	129.4 (4)	H41a—C4a—H43a	109.05
N2a—N1a—C1	119.7 (3)	H42a—C4a—H43a	109.60
N2b—N1b—C3b	111.1 (3)	C1a—C4a—H43a	108.86
C1—N1b—C3b	129.7 (3)	C1b—C4b—H41b	107.16
N2b—N1b—C1	119.2 (3)	C1b—C4b—H43b	112.61
N2c—N1c—C3	119.4 (3)	H41b—C4b—H42b	107.30
C3—N1c—C3c	129.5 (3)	C1b—C4b—H42b	112.56
N2c—N1c—C3c	111.1 (3)	H42b—C4b—H43b	109.56
N2d—N1d—C3	119.7 (3)	H41b—C4b—H43b	107.35
C3—N1d—C3d	129.6 (3)	C1c—C4c—H41c	109.67
N2d—N1d—C3d	110.7 (3)	C1c—C4c—H42c	109.21
Mo1—N2a—N1a	120.4 (2)	H41c—C4c—H42c	109.89
Mo1—N2a—C1a	133.4 (3)	H41c—C4c—H43c	109.61
N1a—N2a—C1a	106.1 (4)	C1c—C4c—H43c	108.91
Mo1—N2b—N1b	121.3 (2)	H42c—C4c—H43c	109.54
Mo1—N2b—C1b	132.7 (3)	C1d—C4d—H42d	110.26
N1b—N2b—C1b	106.0 (3)	C1d—C4d—H43d	110.22
Mo2—N2c—C1c	133.4 (3)	H41d—C4d—H42d	109.01
N1c—N2c—C1c	106.0 (3)	H41d—C4d—H43d	109.04
Mo2—N2c—N1c	120.5 (2)	H42d—C4d—H43d	109.35
Mo2—N2d—N1d	121.1 (2)	C1d—C4d—H41d	108.93
N1d—N2d—C1d	105.9 (3)	C3a—C5a—H51a	110.11
Mo2—N2d—C1d	133.1 (3)	C3a—C5a—H52a	108.24
N1a—C1—N1b	110.4 (3)	H51a—C5a—H52a	110.31
N1b—C1—C2	109.4 (3)	H51a—C5a—H53a	110.34
N1a—C1—C2	110.2 (3)	H52a—C5a—H53a	109.29
N2a—C1a—C2a	109.5 (5)	C3a—C5a—H53a	108.49
N2a—C1a—C4a	122.5 (5)	C3b—C5b—H52b	110.45
C2a—C1a—C4a	128.0 (4)	C3b—C5b—H53b	109.95
N2b—C1b—C2b	109.8 (3)	C3b—C5b—H51b	105.83
N2b—C1b—C4b	122.1 (3)	H51b—C5b—H53b	109.91
C2b—C1b—C4b	128.1 (3)	H52b—C5b—H53b	109.92
N2c—C1c—C4c	121.8 (4)	H51b—C5b—H52b	110.71
C2c—C1c—C4c	128.9 (4)	C3c—C5c—H51c	107.77
N2c—C1c—C2c	109.4 (4)	C3c—C5c—H52c	112.00
N2d—C1d—C2d	110.0 (4)	H51c—C5c—H52c	107.85
N2d—C1d—C4d	121.5 (4)	H51c—C5c—H53c	107.82
C2d—C1d—C4d	128.5 (4)	C3c—C5c—H53c	111.55
O1a—C2—O2a	126.9 (4)	H52c—C5c—H53c	109.66
O1a—C2—C1	114.5 (3)	C3d—C5d—H52d	110.35
O2a—C2—C1	118.6 (4)	C3d—C5d—H53d	110.59
C1a—C2a—C3a	107.3 (4)	C3d—C5d—H51d	108.87
C1b—C2b—C3b	106.9 (4)	H51d—C5d—H53d	108.74
C1c—C2c—C3c	107.5 (4)	H52d—C5d—H53d	109.62
C1d—C2d—C3d	107.5 (4)	H51d—C5d—H52d	108.63

### Hydrogen-bond geometry (Å, °)

**Table:**

*D*—H···*A*	*D*—H	H···*A*	*D*···*A*	*D*—H···*A*
O1w—H1w···O2c	1.02	2.23	2.889 (8)	121
O1w—H2w···Cl2^i^	1.00	2.42	3.335 (7)	151

Symmetry codes: (i) *x*, −*y*+3/2, *z*+1/2.
